# Microdeletion syndromes disclose replication timing alterations of genes unrelated to the missing DNA

**DOI:** 10.1186/1755-8166-2-11

**Published:** 2009-03-14

**Authors:** Josepha Yeshaya, Itay Amir, Ayelet Rimon, Jane Freedman, Mordechai Shohat, Lydia Avivi

**Affiliations:** 1 Raphael Recanati Genetic Institute, Rabin Medical Center Beilinson Campus, Petah-Tikva, Israel; 2 Department of Human Molecular Genetics & Biochemistry, Sackler School of Medicine, Tel-Aviv University, Tel-Aviv, Israel; 3 Department of Pediatrics C, Schneider Children's Medical Center of Israel, Petah Tikva, Israel

## Abstract

**Background:**

The temporal order of allelic replication is interrelated to the epigenomic profile. A significant epigenetic marker is the asynchronous replication of monoallelically-expressed genes versus the synchronous replication of biallelically-expressed genes. The present study sought to determine whether a microdeletion in the genome affects epigenetic profiles of genes unrelated to the missing segment. In order to test this hypothesis, we checked the replication patterns of two genes – *SNRPN*, a normally monoallelically expressed gene (assigned to 15q11.13), and the *RB1*, an archetypic biallelically expressed gene (assigned to 13.q14) in the genomes of patients carrying the 22q11.2 deletion (DiGeorge/Velocardiofacial syndrome) and those carrying the 7q11.23 deletion (Williams syndrome).

**Results:**

The allelic replication timing was determined by *fluorescence in situ hybridization *(FISH) technology performed on peripheral blood cells. As expected, in the cells of normal subjects the frequency of cells showing asynchronous replication for *SNRPN *was significantly (P < 10^-12^) higher than the corresponding value for *RB1*. In contrast, cells of the deletion-carrying patients exhibited a reversal in this replication pattern: there was a significantly lower frequency of cells engaging in asynchronous replication for *SNRPN *than for *RB1 *(P < 10^-4 ^and P < 10^-3 ^for DiGeorge/Velocardiofacial and Williams syndromes, respectively). Accordingly, the significantly lower frequency of cells showing asynchronous replication for *SNRPN *than for *RB1 *is a new epigenetic marker distinguishing these deletion syndrome genotypes from normal ones.

**Conclusion:**

In cell samples of each deletion-carrying individual, an aberrant, reversed pattern of replication is delineated, namely, where a monoallelic gene replicates more synchronously than a biallelic gene. This inverted pattern, which appears to be non-deletion-specific, clearly distinguishes cells of deletion-carriers from normal ones. As such, it offers a potential epigenetic marker for suspecting a hidden microdeletion that is too small to be detected by conventional karyotyping methods.

## Background

Microdeletion syndromes are defined as viable human (constitutional) chromosomal aberrations, each resulting from a small hemizigous loss of DNA (ranging ~1–5 Mb). These losses cannot be detected by conventional karyotyping methods, whose best resolution is no greater than 5 Mb [1,2]. The various microdeletion syndromes are estimated to be among the major causes of mental retardation following Down and Fragile × syndromes [3]. Each is associated with multiple abnormalities in various organ systems, none of which, except for the missing DNA material, is specific or unique to the microdeletion in question, as all occur in the general population as well. However, what makes each syndrome specific and unique is the sum total of the abnormalities observed in each condition, although in the total population of patients each syndrome abnormality is highly variable in its presentation. This is exemplified by the wide spectrum of phenotypic variations characterizing DiGeorge/Velocardiofacial syndrome (DGS/VCFS), and by the variability in expression of Williams syndrome, each of which results from a submicroscopic loss of a tiny chromosomal segment. The large spectrum of anomalies in DGS/VCFS is associated with a deletion in chromosome band 22q11.2, while the variable clinical expression in Williams syndrome results from a deletion in 7q11.23 [4-7]. This permits to put forward the hypothesis that, in addition to the direct impact of the missing DNA, the "tiny monosomic status" by itself may also be implicated in the prenatal malformations and postnatal defects that define the affected phenotype. In view of that, a quantitative imbalance *per se *resulting from the missing DNA may disrupt the normal behavior of other DNA sequences that are present in the aberrant genome in their normal two doses. If, indeed, the loss of one copy of a tiny chromosomal segment can cause a broad spectrum of genomic malfunctions, one should expect to find various non-deletion-specific alterations in the epigenetic properties of various genes (including those situated on chromosomes other than that on which the deletion is situated). To test this hypothesis we examined the temporal order of allelic replication, a marker reflecting epigenetic qualities [reviewed in [8]; see also the next paragraph], of two unrelated genes, *SNRPN *on chromosome 15q11-13 and *RB1 *on chromosome 13q14, in two microdeletion syndromes, DGS/VCFS and Williams syndrome. These two syndromes were chosen because they differ in the missing DNA information, as well as the chromosome on which the missing DNA is located.

A dominant aspect of the inherent epigenomic profile is the exact timing of DNA synthesis at the S-phase of the cell cycle [9-11]. Usually, active loci replicate earlier than silent ones [9,12]. An archetypal example of the close correlation between replication timing and epigenetic inactivity is the difference in replication timing between the active and inactive X-chromosomes in mammalian female cells, whereby the inactive chromosome replicates considerably later than its active counterpart [13,14]. Furthermore, the close connection between replication timing and epigenetic silencing is apparent from the fact that the allelic counterparts of monoallelically expressed genes replicate asynchronously, while the allelic counterparts of biallelically expressed genes replicate synchronously [8,15,16]. A simple method for evaluating the temporal order of allelic replication is the *fluorescent in situ hybridization *(FISH) assay [17,18]. With this assay, it has been demonstrated that allelic counterparts of monoallelically expressed genes show asynchronous replication, with the active allele replicating earlier than the inactive counterpart [18-22]. The asynchronous pattern of allelic replication has been demonstrated in all known types of monoallelically expressed genes. These include: (i) imprinted genes, well exemplified by the *SNRPN *gene located within the Prader-Willi/Angelman syndrome imprinted region, which shows early replication of the active (paternal) allele and late replication of the inactive (maternal) allele [18-22]; (ii) genes subjected to X-chromosome inactivation [23-25]; and (iii) genes undergoing allelic exclusion [8,26-34]. Therefore, the timing of replication of two allelic counterparts (either synchronous or asynchronous), not necessarily in the tissue of expression, is considered to be a marker for the epigenetic properties of the gene in question [30,32-34].

In this study, we have shown in genomes containing a microdeletion, that certain genes, even those unrelated to the deletion, change their characteristic replication properties. Specifically, an imprinted gene (*SNRPN*) reveals relaxation of the asynchronous pattern of replication, characterizing monoallelic expression, whereas genes (*RB1 *and *ARSA*) that normally show synchronous patterns of replication alter their inherent mode and replicate asynchronously. We also found that when a gene replicates asynchronously due to the presence of a deletion, the preference of an allele for early or delayed replication is random.

## Methods

### Subjects and cell cultures

**Figure 1:**
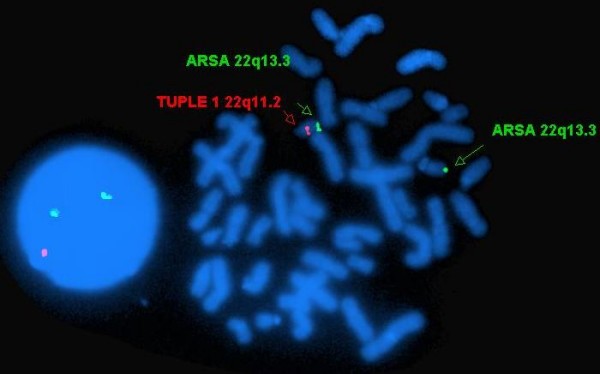
**Cells from a patient with DGS/VCFS, following two-color FISH with *TUPLE1 *(red) and *ARSA *(green)**. Right, a metaphase-spread with the normal chromosome 22, identified by double labeling (red and green signals) and the deletion-carrying one, distinguished by the absence of the red signal; left, an interphase cell exemplifying two *ARSA *(green) signals and a single *TUPLE1 *(red) signal. The green signal that is closer to the red was assumed to belong to the normal homologue, while the more distant one to the deletion-carrying homologue.

Peripheral blood samples obtained from 58 individuals belonging to three different groups of subjects who were referred for cytogenetic testing were used in this study. The first group comprised 30 healthy individuals taken among couples referring to the cytogenetic laboratory due to recurrent pregnancy loss, all of whom were found to have a normal karyotype (designated C1–C30); the second group consisted of 18 patients diagnosed with DGS/VCFS (designated V1–V18), all of whom carried the definitive 22q11.2 deletion confirmed by FISH analysis (Figure 1); and the third group included ten patients with Williams syndrome (cases W1–W10), each of whom carried the characteristic 7q11.23 deletion confirmed by FISH. All the samples were incubated for 72 hours in RPMI 1640 medium supplemented with 20% fetal calf serum (FCS), 3% phytohemagglutinin (PHA) and 1% antibiotics (a standard solution of penicillin, streptomycin and nystatin) at 37°C. Eight control samples (C1–C8), one sample from the DGS/VCFS group (V1) and one sample from the Williams syndrome group (W1) were divided into two cultures, and one culture of each was pulsed-labeled at the S-phase by adding 5'-bromo-2'-deoxyuridine (BrdU; MP Biomedicals, Irvine, CA). At the end of the incubation period, BrdU was added to these cultures at a final concentration of 10^-5 ^M for 90 min at 37°C, followed by a wash with phosphate buffered saline (PBS). Colcemid (Biological Industries, K. Beit-Haemek, Israel) was then added to all the samples, both labeled and unlabeled with BrdU, at a final concentration of 0.1μg/ml for 20 min, followed by hypotonic treatment (0.075-M KCL at 37°C for 15 min) and five washes, each with a fresh cold 3:1 methanol:acetic acid solution.

### Probes

Three directly labeled chromosomal probes from Vysis (Doweners Grove, IL, USA) were used: (i) the *RB1 *probe (32-192018) located on chromosome 13q14; (ii) the *SNRPN *probe (32-190004) located on chromosome 15q11-13; and (iii) the green-labeled *ARSA *probe (32-191028) located on chromosome 22q13.3, which is commercially mixed with the red-labeled *TUPLE1 *probe located on chromosome 22q11.2. In cases with an absence of one copy of 22q11.2, this two-probe combination enabled us, even at interphase, to determine whether an *ARSA *signal was located on the homologue containing the deletion or on the normal partner (Figure 1).

### In-situ hybridization

Each probe was mixed with a hybridization solution and poured onto the fresh slide spreads, covered with a 12 mm cover slip and sealed with rubber cement. Co-denaturation was performed at 76°C for six min followed by 18 hrs hybridization at 37°C in a moist chamber. Following hybridization the slides were washed in 0.4 × SSC/0.3% NP40 at 53°C for 2 min and in 2 × SSC/0.1% NP40 at room temperature for 1 min. After the second washing the slides with the BrdU-labeled samples were incubated with a blocking solution consisting of 1% Bovine serum albumin (BSA, Boerhinger-Mannheim) in 4 × SSC at 37°C for 30 min. At the same time the non-labeled slides were counterstained with DAPI (4,6-diamidino-2-phenylindole, Vector Laboratories Inc., Burlingame, CA) antifade solution and analyzed for simultaneous viewing of FITC, Texas red and DAPI.

### Detection of BrdU-labeled samples

After blocking, BrdU was detected by anti mouse antibody conjugated to AMCA (Jackson Immunoresearch Laboratories, West Grove, PA, USA) linked by mouse anti-BrdU antibody (Dako Cytomation, Glostrup, Denmark). The two antibodies were diluted to the appropriate concentrations in a solution of 4 × SSC with 1% BSA. The slides were incubated with each antibody for 30 min at 37°C, and each incubation was followed by 3 washes in PBS/0.1% Triton × 100 at room temperature for 5 min. After the final washing, the slides were mounted with an antifade solution (Vectashield, Vector Laboratories, Burlingame, CA

### Cytogenetic evaluation

The slides were analyzed blindly on an Olympus BX51 fluorescent microscope fitted with a triple band-pass filter (Chroma Technology, Brattleboro, VT, USA) for the simultaneous detection of three colors: blue-DAPI and AMCA, green-FITC and red-rhodamine or Texas Red.

**Figure 2:**
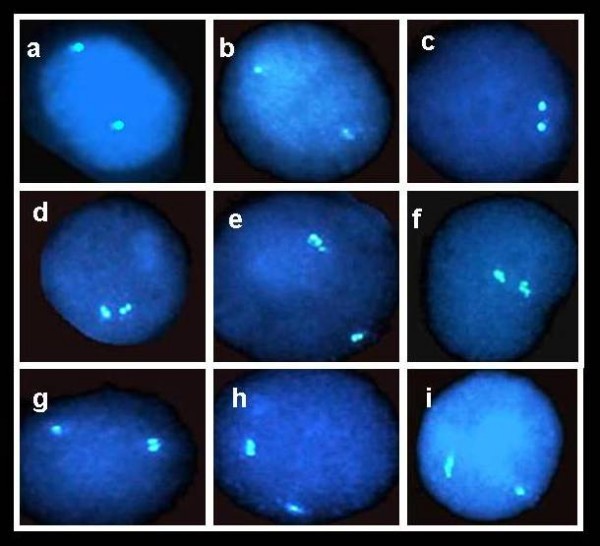
**Fluorescent signals in PHA-stimulated lymphocytes at interphase, following FISH with *RB1***. (a) – (c) cells with two singlets (SS cells) in which neither allele has replicated; (d) – (f) cells with two doublets (DD cells) in which both alleles have replicated; and (g) – (i) cells with one singlet and one doublet (SD cells), which are S-phase cells in which one allele has replicated while its partner has not.

We used the FISH replication assay to determine whether the replication patterns of three loci, *RB1*, *SNRPN *and *ARSA*, in samples of control individuals and patients with microdeletion syndromes were synchronous or asynchronous. For each case, each probe and each BrdU-labeled and unlabeled sample we analyzed at least 100 interphase cells that showed, following FISH, two clearly stained hybridization signals (Figure 2). We noted the structure of each fluorescent signal in these cells – i.e. whether it was a singlet (S), representing a non-replicated sequence, or a doublet (D), indicating a replicated sequence. Accordingly, the cells were classified into three categories: (i) cells with two singlets (SS cells; Figure 2a, b, c); cells with two doublets (DD cells; Figure 2d, e, f); and (iii) cells with one singlet and one doublet (SD cells; Figure 2g, h, i). Thus, in a population of replicating cells, following hybridization with a locus specific probe, the frequency (%) of cells containing two signals differing in their replication status (SD cells), out of the total population of cells with two hybridization signals, represents the level of asynchrony in the replication timing of the allelic counterparts of the locus in question.

In order to differentiate in the interphase cell samples of the DGS/VCFS patients between the *ARSA *locus located on the deletion-carrying homologue and the one situated on the normal chromosome, we used the signal of the *TUPLE1 *allele as a reference point. We assumed that the *ARSA *allele that is closer to the *TUPLE1 *allele is located on the normal chromosome whereas its counterpart is located on the chromosome carrying the deletion (Figure 1).

### Statistical method

The statistical significance of the differences between the two cell populations tested was determined by the two-tailed Student's *t*-test.

### Ethical basis

The study was approved by the Rabin Medical Center Review Board

## Results

**Figure 3:**
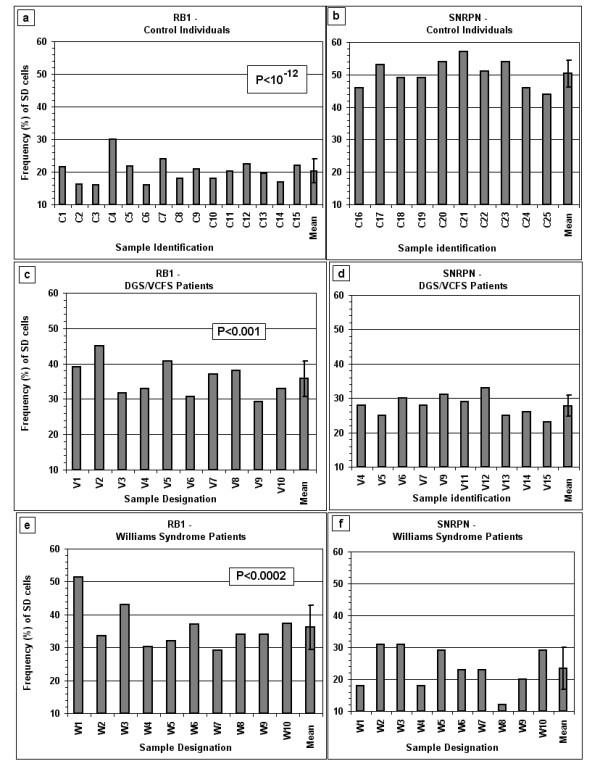
**The frequency of SD cells for *RB1 *and *SNRPN***. (a) and (b), samples from control individuals; (c) and (d), samples from patients with DGS/VCFS; and (e) and (f), samples from patients with Williams syndrome. The P values in frames (a), (c) and (e) represent the level of significance of the differences in the frequency of SD cells between *RB1 *and *SNRPN *within a given group of samples. The mean frequency and standard deviation for each locus in each group of samples are also shown (last bar in each frame).

The frequency of SD cells for the *RB1 *locus in PHA-stimulated lymphocytes of control subjects (cases C1–C15) ranged from 16% to 30% with a mean and standard deviation value of 20.3 ± 3.7% (Figure 3a). Specifically, in 14 out of the 15 control subjects tested the *RB1 *locus showed a low frequency of SD cells (less than 25%) (Figure 3a). In contrast, as expected for an imprinted locus, in a group of 10 control subjects (cases C16–C25) the frequency of SD cells for the *SNRPN *locus was almost twice as high as that found for the *RB1 *locus, ranging from 44% to 57% with a mean and standard deviation value of 50.3 ± 4.2% (Figure 3b). Clearly, in cells of control subjects the imprinted *SNRPN *locus shows significantly higher frequencies of SD cells compared to *RB1 *(P < 10^-12^).

However, in the cell samples from the DGS/VCFS and the Williams syndrome patients, the frequency of SD cells for the imprinted *SNRPN *locus were significantly lower than the corresponding values for *RB1 *(P < 0.001 for the DGS/VCFS patients and P < 0.0002 for those with Williams syndrome; Figure 3c, d, e, f). Specifically, the frequency of SD cells for *SNRPN *in the samples from the DGS/VCFS patients ranged from 23% to 33% with a mean of 27.8 ± 3.1%, and the corresponding *RB1 *values ranged from 29% to 45% with a mean of 35.7 ± 5.0% (Figure 3c and Figure 3d). Similarly, the frequency of SD cells in the samples from the Williams syndrome patients ranged from 12% to 31% for the *SNRPN *locus and from 29% to 51% for the *RB1 *locus with a mean of 23.4 ± 6.5% and 36.2 ± 6.7%, respectively (Figure 3e and Figure 3f).

**Figure 4:**
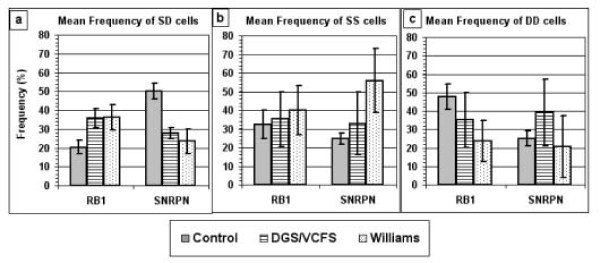
**Mean frequency values for SD, SS and DD cells for *RB1 *and *SNRPN *in control and patient samples**. Solid bars present the control sample (cases C1–C15 for *RB1 *and C16–C25 for *SNRPN*); striped bars present the sample of patients with DGS/VCFS (cases V1–V10 for *RB1*, and V1, V5–V7, V9 and V11–V15 for *SNRPN*); and dotted bars present the sample of patients with Williams syndrome (cases W1–W10).

The considerably low frequency of SD cells for *SNRPN *found in the samples of the two groups of patients with the deletion syndromes were similar to each other (P > 0.05), but each deviated significantly from those characterizing the samples of the control group (P < 10^-9 ^and P < 10^-7^, for DGS/VCFS and Williams syndromes, respectively) (Table 1; Figure 3b, d and Figure 3f; Figure 4a). The samples of the two groups with the deletion syndromes also showed similar SD frequencies for *RB1 *(P > 0.80), which were significantly higher than the corresponding *RB1 *value observed in the control group of samples (P < 10^-6 ^and P < 10^-4^, for DGS/VCFS and Williams syndromes, respectively) (Table 1; Figure 3a, c and Figure 3e; Figure 4a).

**Table 1:** Level of significance (P) of the differences in corresponding SD, SS and DD values between the designated groups

	**SD**		**SS**		**DD**	
	
	*RB1*	*SNRPN*	*RB1*	*SNRPN*	*RB1*	*SNRPN*
	
Control vs. DGS/VCFS	P < 10^-6^	P < 10^-9^	P > 0.50	P > 0.10	P < 0.05	P < 0.05
Control vs. Williams	P < 10^-4^	P < 10^-7^	P > 0.05	P < 0.0005	P < 10^-4^	P > 0.40

DGS/VCFS vs. Williams	P > 0.80	P > 0.05	P > 0.40	P < 0.01	P > 0.05	P < 0.05

Control group (cases C1–C15 for *RB1 *and C16–C25 for *SNRPN*); DGS/VCFS group (cases V1–V10 for *RB1*, and cases V1, V5–V7, V9, V11–V15 for *SNRPN*); and Williams syndrome group (cases W1–W10).

**Figure 5:**
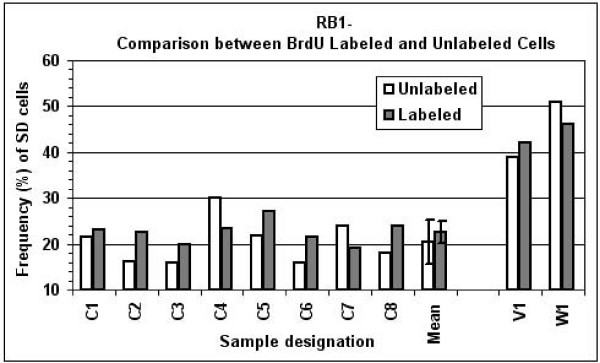
**Frequency of SD cells for *RB1 *in BrdU-labeled and unlabeled cell populations**. Control individuals (cases C1–C8), a patient with DGS/VCFS (case V1) and a patient with Williams syndrome (case W1). Each sample is shown with two SD values for *RB1*, one in an unlabeled cell population (open bars) and the second in a BrdU labeled population (solid bars).

When the SD frequencies for *RB1 *were analyzed in distinguishable (BrdU-labeled) S-phase cell populations from eight control subjects (cases C1–C8) and two patients (cases V1 and W1), it was seen that each of the two cases with the deletion syndrome revealed in the BrdU-labeled S-phase cell population a high frequency of SD cells for *RB1 *(42% and 46%, for V1 and W1, respectively), similar to the corresponding value obtained in the unlabeled S-phase cell population (Figure 5). Also, each of the control samples tested showed, for *RB1*, a frequency of SD cells in the S-phase labeled cell population similar to the corresponding value obtained in the unlabeled cell population (less than 24%) (Figure 5).

As judged from the frequencies of the SS- and DD- cells for *RB1 *in the three groups of samples tested, it appears that the increased frequency of SD cells for *RB1 *in the patients with a deletion syndrome results from delay in replication of a single *RB1 *allele. Specifically, in each group with a deletion syndrome, the frequency of SS cells for *RB1 *was similar to the corresponding value in the control group. However, the frequency of DD cells in each group with a deletion syndrome was significantly lower than the corresponding value in the control group (Table 1; Figure 4b and Figure 4c).

When analyzing the frequencies of the SS and DD cells for *SNRPN*, it appears that the decreased frequency of SD cells observed in the DGS/VCFS patients is accompanied by a statistically significant increase in the frequency of DD cells but not of SS cells, while the decreased frequency of SD cells observed in the Williams syndrome patients is associated with an increase in the frequency of SS cells but not of DD cells (Table 1; Figure 4b and Figure 4c).

**Figure 6:**
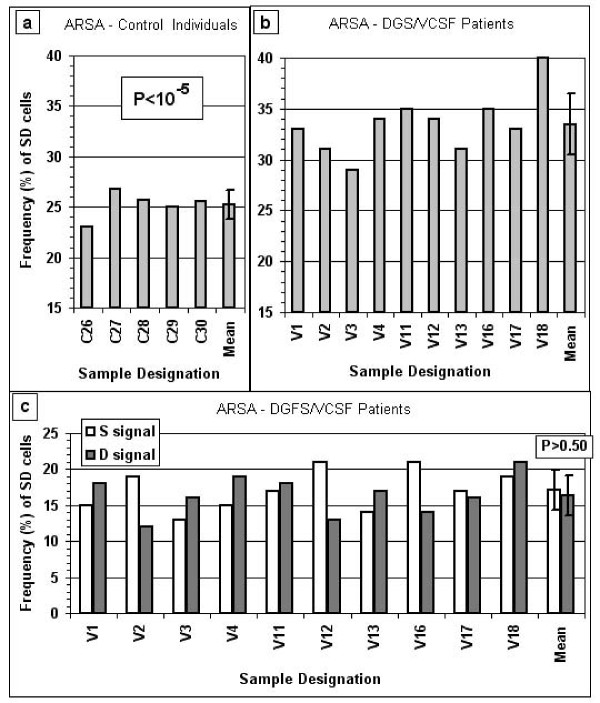
**Frequency of SD cells for *ARSA *in samples from control individuals and from patients with DGS/VCFS**. In frame (c) the frequency of SD cells in each individual sample was separated into two sub-categories: cells in which the *ARSA *signal of the deletion-carrying homologue shows a singlet (S-signal, open bar) and those in which the *ARSA *signal of the deletion-carrying chromosome reveals a doublet (D-signal, solid bar). The mean frequencies and standard deviations for each group of samples are also given (last bar(s) in each frame). The P value in frame (a) represents the significance of the difference between the values in frames (a) and (b); the P value in frame (c) shows the significance of the difference between the S and D values for the *ARSA *signal of the deletion-carrying homologue within the total *ARSA *SD cell population.

Finally, in order to determine whether proximity to a deleted region affects the replication timing properties of an adjacent locus we studied the replication mode of the *ARSA *alleles in cell samples from ten DGS/VCFS patients (cases V1–V4, V11–V13 and V16–V18) and five control subjects (cases C26–C30) (Figure 1). We found that the frequency of SD cells for the *ARSA *locus in the samples from the patients was significantly higher than that observed in the control samples (P < 10^-5^) (Figure 6a and Figure 6b). It appears that the *ARSA *locus mimics the behavior of the *RB1 *locus, exhibiting SD frequencies in control samples that were significantly lower than the corresponding *SNRPN *values (P < 10^-9^), and levels in samples from subjects carrying a microdeletion that were significantly higher than the corresponding *SNRPN *values (P < 0.0003). However, the replication timing behavior of the *ARSA *allele located on the homologue carrying the deletion was similar to that of its counterpart situated on the normal chromosome. Therefore, it appears that the *ARSA *allele on the homologue with the deletion, as compared to its counterpart, showed neither a greater tendency for earlier replication (D signal) nor an increased preference for delayed replication (S signal). This shows that in each SD cell population of the ten DGS/VCFS cases studied, the preference for early or late replication of the *ARSA *allele located on the deletion-carrying homologue was random (P > 0.50; Figure 6c).

## Discussion

Our results are in accordance with data showing that the *SNRPN*-imprinted locus – the hallmark of monoallelic expressed genes – normally exhibits asynchronous replication [8,30,32]. It has also been well documented that the expressed allele, which in the case of the *SNRPN *gene is the paternal allele, replicates earlier than its counterpart [16,20]. Clearly, the *SNRPN *pattern of replication differs markedly from the common synchronous pattern characterizing most genes in the human genome – namely, the biallelically expressed genes [12], exemplified here by *RB1 *and *ARSA*.

However, in the cells of microdeletion syndrome patients (of 22q11.2 or 7q11.23), the *SNRPN *gene revealed a loss of its asynchronous pattern of replication. In addition, in cells of the patients, the *RB1 *and the *ARSA *loci also exhibited replication-timing alterations, that is, both these genes demonstrated an asynchronous replication pattern. Consequently, an abnormal epigenetic profile is delineated inside genomes carrying a deleterious microdeletion, in which allelic counterparts of an imprinted locus replicate more synchronously than allelic counterparts of biallelically expressed loci.

It is clear that the considerably low frequency of SD cells for *SNRPN *in the deletion syndrome cases we studied cannot be attributed to a shortening of the duration of the S-phase, since there was a concomitant, significant increase in the frequency of SD cells for *RB1*. On the other hand, while an increase in the S-phase duration may explain the increase in the frequency of SD cells for *RB1*, it fails to explain the dramatic decrease in the frequency of SD observed for *SNRPN*. Furthermore, the S-phase-labeled cell population revealed similar values for *RB1 *to those obtained in the unlabeled cell population. Thereby, indicating directly that the duration of the S-phase was not associated with an alteration in the replication timing accompanying the deletion syndromes.

Since the loci studied here were selected at random, unrelated to the deletions in question, we hypothesize that other monoallelically expressed loci (in addition to *SNRPN*), as well as other biallelically expressed ones (in addition to *RB1 *and *ARSA*), in these genomes may also exhibit replication timing alterations. Considering the strong correlation between replication timing and epigenetic characteristics, it is reasonable to assume that the global replication timing modifications displayed within the deletion-carrying genomes lead to major alterations in the epigenomic features. This reasoning is in line with the view that the replication timing is both the cause and the consequence of the chromatin structure of a gene [9]. We have shown here that alleles of a gene that is normally subject to monoallelic expression fail to keep the inherent functional asymmetry between the allelic counterparts necessary for establishing normal phenotypes. Conversely, the normally biallelically expressed loci fail to keep the functional symmetry between parental counterparts required to fulfill Mendelian laws. This loss of symmetry in deletion-carrying genomes for alleles of biallelically expressed genes co-occurring with loss of the inherent capability of exclusively specialized genes for allele-specific behaviour, may explain the numerous and variable, non-deletion-specific abnormal traits that accompany both of these microdeletion syndromes.

Interestingly, in the DGS/VCFS group the loss of replication asynchrony observed for the *SNRPN *locus is mainly due to an advanced replication of the normally late-replicating (maternal) allele, while the loss of asynchrony for *SNRPN *in the Williams syndrome patients is mostly associated with a delay in the replication timing of the normally early-replicating (paternal) *SNRPN *allele. Yet, it is illogical to assume that the deletion in question "recognizes" whether a far-removed allele is maternal or paternal in origin through some parent-of-origin inborn signal. The differentiation is possibly based on the epigenetic differences characterizing alleles of the imprinted locus that were established in the parental germ cells prior to fertilization [35]. Whatever the reason for the differences in *SNRPN *replication behavior in the two deletion syndromes, it clarifies how loss of asynchronous replication, even for a single and specific monoallelic gene, may lead (in addition to non-deletion-specific malformations) to syndrome-specific abnormal phenotypic expression. It is interesting to note that relaxation of imprinting, arising either from activation of the silent allele or from silencing of the normally active allele was documented previously in association with the numerous, variable epigenetic alterations characterizing malignant phenotypes [36].

One could speculate, therefore, that each microdeletion shows specificity toward a particular epigenetic profile, and if the two parental allelic counterparts retain the same structure, they are not differentiated specifically by the deletion in question. This view is in accordance with the replication pattern of the *ARSA *locus, which replicates synchronously in normal cells, and as such its alleles are expected to have initially (at fertilization) similar epigenetic structures. Indeed, our results show that in cells carrying the 22q11.2 deletion, the asynchronous replication of the *ARSA *locus resulted from a random preference of an allele for early or late replication, probably determined *de-novo *for each replication cycle. This is inferred from the observation that in half (about 50%) of each population of SD cells from patients with the 22q11.2 deletion, the allele located on the homologue carrying the deletion was the early-replicating allele, while in the other half its counterpart was the early one. Additionally, it indicates that an allele located distally to the deleted region, on the same chromosomal arm, behaves similarly to its counterpart located on the normal arm. Together, our results disagree with the notion that asynchronous replication in a deletion-carrying genome results merely from a type of position-effect (change in position along the chromosome) of the allele located on the deletion-carrying homologue [37]. Our results are in agreement with a short report claiming that the presence of a microdeletion within the genome increases the level of replication asynchrony of biallelically expressed genes not located on the deletion-carrying chromosome [38]. Furthermore, we show here that the asynchronous pattern of replication of biallelically expressed loci, exemplified by *RB1 *in cells with a 22q11.2 deletion, as well as those with a 7q11.23 deletion, appears to result from a delay in replication of a single allele (inferred by a decrease in the frequency of DD cells but not of SS cells). Yet, based on the replication pattern of the *ARSA *locus, which mimics the replication behavior of the *RB1 *locus, it is reasonable to assume that in a deletion-carrying genome, the preference of a specific allele of a biallelically expressed gene for delayed replication is also random. It is therefore independent of any kind of position effect. This is supported by a study in cells with a 22q11.2 deletion that the *TUPLE1 *allele, situated on the intact chromosome 22 within the 22q11.2 region, presents a delayed replication timing [6].

Asynchronous replication of biallelically expressed loci has been reported in cells from patients carrying an aneuploidic loss of the whole X-chromosome (Turner syndrome, shown in peripheral blood cells) [39] or the gain of an autosome (trisomies 21, 18 and 13, shown in amniotic fluid cells) [40,41]. In these whole-chromosome aneuploidies [39-41], the biallelically expressed loci that were shown to change their characteristic synchronous pattern of replication to an asynchronous one were not associated with the lost or gained chromosome. Taken together, it is reasonable to assume that the phenomenon of replication-timing alteration in loci unrelated to the imbalance-causing-DNA sequence is not specific to the size or type of the lost/gained chromosomal segment (or whole chromosome). Thus, the view that a specific gene(s) assigned to the missing segment, in each of the microdeletion genotypes studied here, is the sole cause of the global replication timing aberrations is weakened. This reasoning remains coherent even in light of the information that the deletion of the DGS/VCFS is gene-dense [reviewed in [6]] and the deletion of the Williams syndrome carries a gene (*RFC2*) that codes for a subunit of a replication factor, a part of the multimeric complex involved in DNA elongation during replication [7]. Furthermore, even the view that replication-timing alterations are associated with an existing predisposition factor that destabilizes the integrity of the genome appears to be incompatible with the current data; any potential destabilizing mechanism leading to a segmental deletion/duplication [4,5,42,43] is entirely different from that giving rise to the loss/gain of a whole chromosome [44]. Therefore, it is more logical to assume that the global alteration in allelic replication timing results from the aneuploidic status itself. This is in accordance with data showing that peripheral blood cells derived from individuals with normal karyotypes, which show increased levels of sporadic (non-chromosome specific) aneuploidy due to malignancy, exhibit replication timing modification of various loci unrelated to the missing/gained chromosomes [45-47]. This further strengthens the notion that the nature of the missing/gained genes assigned to the lost/gained DNA is not imperative to the replication anomaly described here.

Yet, the non-locus-specific aberrant replication timing in the cells of cancer patients was reverted to normal in the presence of 5-azacytidine, a classical methylation-blocking agent, recently approved as an anti-cancer drug [48], thereby linking replication timing alteration to methylation capacity [46,47,49]. This agrees with our previous studies in cells of mutation carriers [25] and patients with the fragile × syndrome [50], where we showed that the mutated (*FMR1*) allele (characterized by highly methylated tri-nucleotide repeats) demonstrated delayed replication, which was restored by the application of a demethylating drug [50]. Similarly, replication studies in cells of girls with Rett syndrome link loss of asynchronous replication of X-linked genes with DNA methylation [51]. Rett syndrome is caused by a mutation in an X-linked gene (*MECP2*) that normally codes for a protein capable of binding to methylated DNA; it therefore takes part in the line of events leading to transcriptional silencing of X-inactivated genes [reviewed in [52]].

The involvement of methylation capacity with replication timing is not surprising since allelic counterparts, which exhibit normally asynchronous replications are most often differentially methylated [8,12]. It is therefore reasonable to assume that also the aberrant replication accompanying a microdeletion is associated with aberrant methylation. It is yet not clear if independent mechanisms control allelic replication timing (synchronous or asynchronous) and methylation capacity (similar or different). Nevertheless, the non-random spatial organization of homologous DNA segments in the diploid nucleus was suggested to play a critical role in both replication timing and methylation capacity [8,12]. Furthermore, numerous studies also show that DNA spatial organization in the diploid nucleus is most crucial for epigenomic stability and proper gene functioning [reviewed in [53]]. Therefore, it is tempting to speculate that the loss/gain of a single chromosomal segment (or whole chromosome) disrupts the structural unity of the diploid status, altering the overall spatial organization of chromosomes within the interphase nucleus. This in turn leads to (non-gene specific) epigenetic alterations of genes situated on chromosomes other than those carrying the aberration, as well as those located on the aberrant chromosome. This speculation is supported in part by data showing that in cells of DGS/VCFS patients the intact chromosome 22 displays a specific (peripheral) nuclear location, which is different from that observed in normal cells, for two intact (fully homologous) chromosomes 22 [6].

Whatever the mechanism underlining abnormal allelic replication timing, the epigenetic alteration delineated by the deletion is not deletion-specific, and probably accompanies any loss/gain of a whole chromosome or of a small chromosomal segment. As such, abnormal allelic replication timing offers a potential epigenetic marker, a kind of a preliminary hint to suspect the existence of any type of chromosomal imbalance. For instance, the replication-timing test can be used to investigate unexplained cases of mental retardation as well as other phenotypic abnormalities suspected to arise from minute segmental aneuploidy that cannot be seen by conventional karyotyping [1,54]. Specifically, cases showing a higher level of asynchrony for a biallelic gene compared to that observed (in the very same cell sample) for a monoallelic one, call for further investigations. Moreover, the global epigenetic alteration, as described here in PHA-stimulated lymphocytes of microdeletion-carrying patients, probably appears in prenatal tissues as well, as can be inferred from the high asynchronous replication of biallelically expressed loci observed previously in the amniotic fluid cells of cases with trisomies 21, 18 and 13 [40,41]. It is therefore reasonable to assume that observation of a reversal of the replication patterns in amniotic cells might mark a fetus that has inherited a chromosomal imbalanced genome and thus, it calls for further attention. One could seek to apply more laborious and costly DNA analyses for examining the integrity of the entire chromosomal complement. These might include high-resolution array-based comparative genomic hybridization techniques [2,3,54], capable of characterizing the specific DNA segment incriminated in a chromosomal imbalance.

Finally, we would like to refer shortly to the abundance of submicroscopic copy number variations (CNV) of DNA segments ranging from a few kilobases (kb) to megabases (Mb) in size recently discovered in the human genome, whose impact upon the genome is still undefined [55,56]. It is worth examining whether those variations are accompanied by replication-timing alterations and whether such alterations could be of use in differentiating between deleterious and harmless CNVs.

## Conclusion

Within genomes carrying a deleterious microdeletion, an abnormal epigenetic profile is delineated in which allelic counterparts of an imprinted gene, not directly associated with the missing DNA, replicate more synchronously than allelic counterparts of biallelically expressed loci. This epigenetic aberration, which appears to be non-deletion specific, is in line with the numerous non-deletion specific phenotypic abnormalities usually accompanying each microdeletion syndrome. Such an aberration, which is easily detected by cytogenetic means, offers a potential preliminary screening tool for such conditions, in effect, an "epigenotype-first approach" that can be performed without any prior information as to the specific genetic nature of the underlying abnormality.

## Competing interests

The authors declare that they have no competing interests.

## Authors' contributions

JY and IA have contributed equally to this work. Both, participated in the design of the study, carried out most of the cytogenetic work, performed the statistical analyses and helped to draft the manuscript. AR and JF carried out some of the cytogenetic studies and participated in the collection of samples. MS was involved in the design of the study. LA conceived of the study, participated in its design, and coordinated and drafted the manuscript. All authors read and approved the final manuscript.
